# Xanthogranulomatous Endometritis: A Report of Two Cases

**DOI:** 10.7759/cureus.38226

**Published:** 2023-04-27

**Authors:** Christian Silva-Rengifo, Angelica Asencio, Oscar Salirrosas

**Affiliations:** 1 Department of Obstetrics and Gynecology, Hospital Nacional Guillermo Almenara Irigoyen, Lima, PER; 2 School of Medicine, Universidad Nacional Mayor de San Marcos, Lima, PER; 3 Department of Pathology, Hospital Nacional Guillermo Almenara Irigoyen, Lima, PER; 4 Department of Surgery, St. Elizabeth's Medical Center, Boston University School of Medicine, Boston, USA

**Keywords:** inflammatory, histiocytic endometritis, endometrial carcinoma, xanthogranulomatous inflammation, xanthogranulomatous endometritis

## Abstract

Xanthogranulomatous endometritis (XGE) or histiocytic endometritis is a chronic inflammatory pathology of rare presentation, characterized by an exaggerated inflammatory infiltrate that can mimic an endometrial carcinoma. We report two cases of this disease, one of them with a classic presentation of endometritis and the other one with a severe compromise in which the clinical presentation and imaging findings suggested a possible endometrial carcinoma. Knowledge of this unusual and rare pathology, including its etiopathogenesis, is important since it can be included in the differential diagnosis of endometrial carcinoma and, therefore, whenever it is found, to avoid excessive treatment.

## Introduction

Xanthogranulomatous inflammation is an uncommon type of chronic inflammation characterized by an exaggerated infiltration of inflammatory cells, such as foamy cells, among others, in the affected tissues, simulating a local neoplastic invasion. This pathology has been reported mostly in the kidney and gallbladder [1,2], being the involvement of the female genital tract a rare finding. Although it is a very well-described lesion, it continues to remain unfamiliar to clinicians and pathologists due to its infrequency, and its incidence has not been established because of its rarity [3].

Xanthogranulomatous endometritis (XGE), also known as histiocytic endometritis, is an unusual endometrial disease that can represent a diagnostic challenge due to its ability to mimic endometrial carcinoma [4], and to date, only a few cases have been reported.

The confirmatory diagnosis of this pathology is made with a histopathological study. It is characterized by the presence of foamy histiocytes, plasma cells, lymphocytes, neutrophils, necrosis, and fibrosis in the endometrium [5].

In this opportunity, we present two cases of XGE. In one of them, the uterine wall was completely compromised, with the inflammatory process extending beyond the serosa, with CT images suggestive of bladder infiltration.

## Case presentation

Case 1 (2019)

A 65-year-old Peruvian female presented to the gynecology office referring to a two-year history of postmenopausal bleeding accompanied by pain and abdominal distension. The patient had a previous diagnosis of genital dystopia (grade III cystocele, rectocele, and ureterocele). Otherwise, she had no other chronic medical conditions, previous surgeries, or a history of hormone replacement therapy.

**Figure 1:**
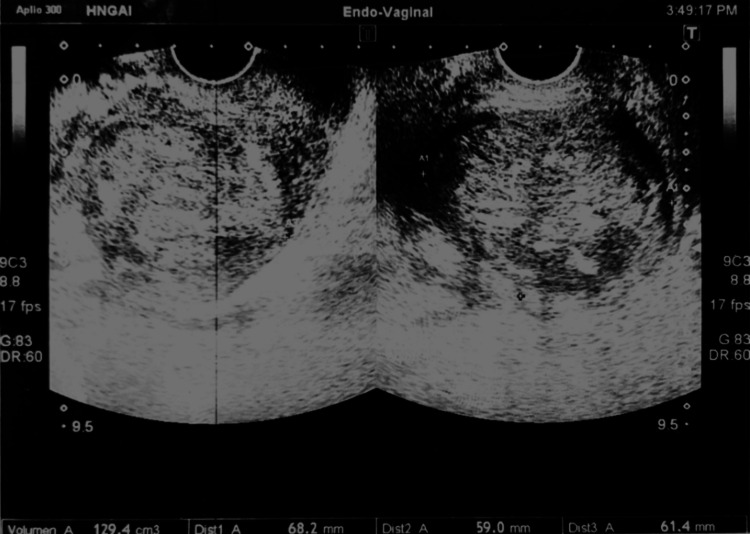
Transvaginal ultrasound: heterogeneous solid tumor, with smooth and well-defined contours, apparently encapsulated, that occupies the uterine body and fundus It measures 68.2 × 59 × 61.4 mm with a volume of 129.4 cc.

A decision was made to start imaging studies with a transvaginal ultrasonography that showed an apparently encapsulated, heterogeneous solid tumor with well-defined borders, occupying the uterine corpus and fundus (Figure 1). The tumor measured 68 × 59 × 62 mm with a volume of 129.4 cc. There was no free fluid or intra-abdominal collections. Doppler US revealed a resistance index (RI) of 0.62 and a pulsatility index (PI) of 0.97. The US impression stated a possible uterine sarcoma or a uterine myoma.

The abdominopelvic CT scan stated the following: (1) tumor-like thickening of the walls of the vagina and anterior wall of the uterus that shows apparent contact with the posterior wall of the bladder (cannot rule out possible bladder neoplasm) and is also associated with mild inflammatory changes in the peri-uterine fat, (2) extensive heterogeneous solid tumor of the left adrenal gland suggesting probable metastasis, and (3) presence of 25-mm lymph nodes in the right external iliac chain and 20-mm lymph nodes in the left external iliac chain.

Relevant laboratory workup showed normal hematocrit (33%), leukocytosis (WBC of 15,160/µL with a WBC differential including 16% bands, 73% neutrophils, 4% lymphocytes, and 7% monocytes), elevated C-reactive protein (CRP) (162.9 mg/L), and negative results for tumor markers carbohydrate antigen (CA) 19-9 (5.51 U/mL), carcinoembryonic antigen (CEA) (<0.50 ng/mL), alpha-fetoprotein (AFP) (<1.3 ng/mL), beta-human chorionic gonadotropin (β-hCG) (<2 mUI/mL), and cancer antigen 125 (CA-125) (5.9 U/mL).

Two endometrial biopsies were taken, revealing fibromuscular tissue with chronic inflammatory infiltrate, with no evidence of endometrial tissue. Additionally, a cystoscopy showed no evidence of malignancy.

The patient was scheduled for an exploratory laparotomy. A hysterectomy + bilateral salpingo-oophorectomy + left adrenalectomy was performed.

**Figure 2:**
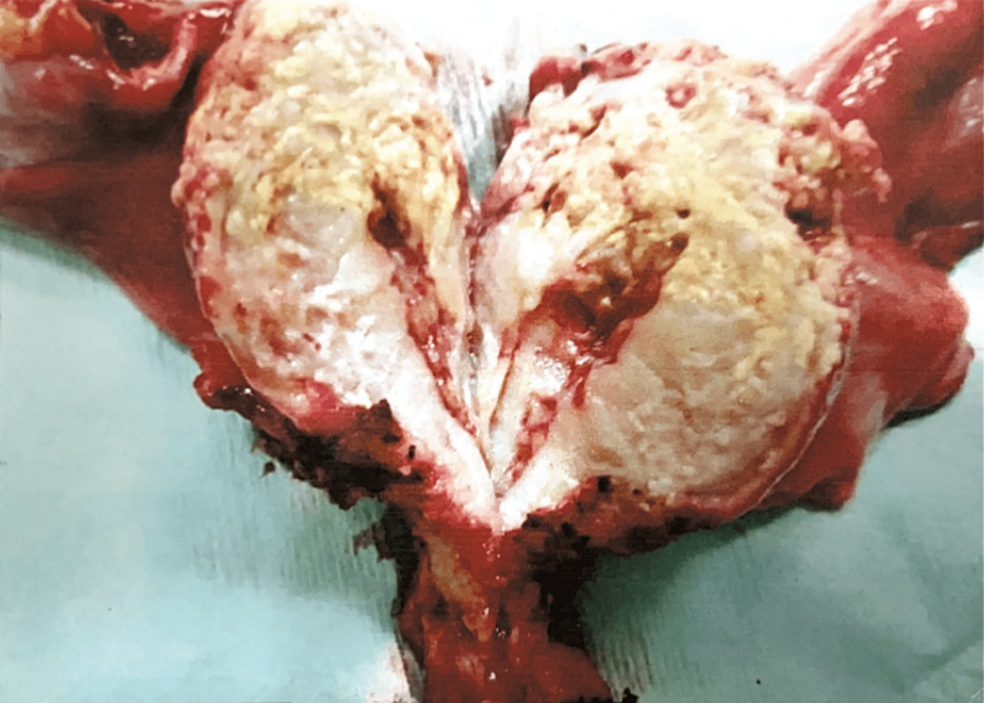
Pathological specimen demonstrating dilated uterine cavity and thickened endometrium covered with a yellowish material

On gross examination, the endocervical canal was obstructed by an internal cervical os stenosis, the uterine cavity was dilated, and the endometrium was thickened and lined with a friable, yellowish material. Likewise, the myometrium was found thickened at the level of the uterine fundus with a yellowish tumor-like lesion that exceeded the serosa in that area (Figure 2).

**Figure 3:**
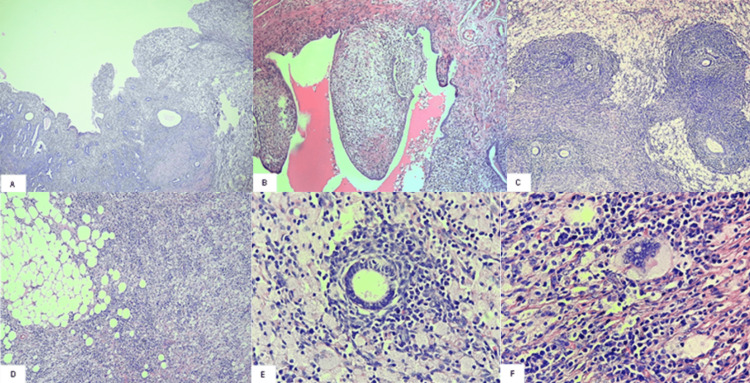
Histopathological findings of the uterus and omentum (A) Photomicrograph showing the replacement of the endometrium (on the right side) by sheets of foamy histiocytes infiltrating the myometrium (H&E: 4×). (B) Photomicrograph showing the presence of foamy histiocytes in the lamina propria of the uterine tube (H&E: 4×). (C) Photomicrograph showing the presence of foamy histiocytes surrounding foci of adenomyosis (H&E: 10×). (D) Photomicrograph of the omentum infiltrated by xanthogranulomatous inflammation (H&E: 10×). (E) Photomicrograph showing foamy histiocytes, lymphocytes, and plasma cells at higher magnification, as well as the remaining endometrial gland (H&E: 40×). (F) Photomicrograph showing foamy histiocytes, lymphocytes, plasma cells, and a multinucleated giant cell (H&E: 40×). H&E: hematoxylin and eosin

The pathology report revealed the following: (1) the endometrium was replaced by sheets of foamy histiocytes mixed with plasma cells, polymorphonuclear cells, and few preserved endometrial glands; no endometrial hyperplasia or carcinoma was found; the sections made at the level of the uterine fundus showed that the inflammatory infiltrate observed in the endometrium invaded the entire myometrium and surpassed the serosa; and the said infiltrative pattern of histiocytic inflammation was associated in foci of adenomyosis; (2) the aforementioned findings were observed also in the uterine tubes, associated with foci of tubal endometriosis; and (3) left suprarenal tumor (adrenal myelolipoma) (Figure 3).

A week later, in her control appointment at the gynecology office, no symptoms or signs of systemic infection were evident, and the laboratory results were within normal parameters. The patient was discharged, and future appointments were scheduled for subsequent outpatient checkups.

Case 2 (2022)

An 84-year-old postmenopausal Peruvian female presented to the emergency department (ED) with a history of malaise and greenish vaginal discharge that began several months ago. Her prior medical history included rheumatoid arthritis and osteoporosis, with no previous surgical procedures. She denied symptoms such as weight loss, hyporexia, vaginal bleeding, or pelvic pain.

A speculoscopy was performed, and it revealed a large amount of greenish discharge for which an abdominopelvic CT scan was requested.

**Figure 4:**
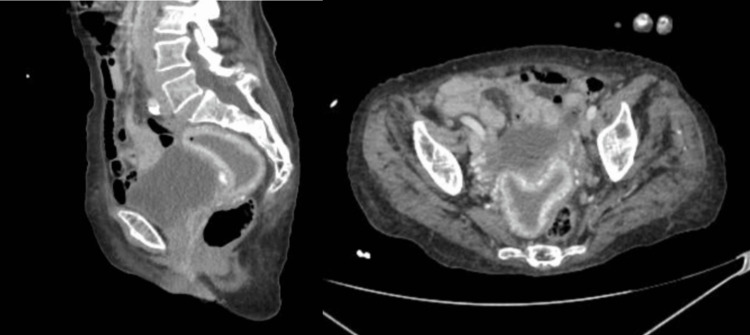
CT scan: endometrial cavity with hypodense content showing the presence of air bubbles and myometrial hyperemia CT: computed tomography

The CT scan report stated dilatation of the endometrial cavity, gas and dense liquid (pyometra was considered), and association with thickening of the cervix (tumor lesion or cervicitis cannot be ruled out), as well as striation of the adjacent mesenteric fat and inflammatory thickening of the peritoneal folds of the pouch of Douglas and the posterior bladder wall (Figure 4). Direct exploration is suggested.

Back in the ED, the uterine cavity was drained with a 10-French Nelaton catheter. Approximately 60 cc of foul-smelling greenish discharge was obtained and sent for microbiological examination.

Laboratory studies were notable for a mild leukocytosis (WBC of 11,540/µL with a WBC differential including 1% bands, 84% neutrophils, 9% lymphocytes, and 6% monocytes), elevated C-reactive protein (71.6 mg/L), and tumor markers within normal ranges (CEA: 1.95 ng/mL, CA 19-9: 3.72 U/mL, CA-125: 59.56 U/mL, and AFP: 2.99 ng/mL).

The patient was hospitalized, and antibiotics treatment was indicated (metronidazole 500 mg IV every eight hours and ciprofloxacin 400 mg IV every 12 hours). In the context of an elderly female with pyometra and vaginal discharge, the possibility of endometrial cancer could not be ruled out at this point.

During her hospitalization, a hysteroscopy was performed under epidural anesthesia. Yellowish-white secretion with a foul odor was evident in a regular quantity; approximately 150 cc of that secretion was sampled and sent to culture. Endocervical and endometrial curettage was made, as a biopsy of endometrial tissue, and the samples were sent for anatomopathological study.

**Figure 5:**
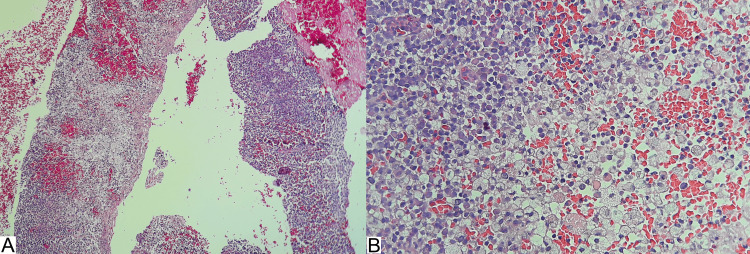
Endometrial biopsy (A) Photomicrograph showing the endometrial biopsy obtained (H&E: 4×). (B) At higher magnification, the presence of foamy histiocytes, lymphocytes, plasma cells, and red blood cells is observed (H&E: 10×). H&E: hematoxylin and eosin

The pathology report revealed sheets of foamy histiocytes, plasma cells, and lymphocytes. No endometrial glands were observed (Figure 5).

After the procedure, there were no systemic warning signs, her vital functions were within adequate parameters, and the speculoscopy did not show leukorrhea or vaginal bleeding. She was discharged, and an appointment was made for follow-up at the outpatient gynecology clinic. No further surgical treatment was necessary.

## Discussion

This presentation of XGE is very uncommon and mimics endometrial cancer very well. The first case report was reported by Barua et al. [6] in 1978 in the Indian population. This case series is the first report on the literature on the Peruvian population.

Although xanthogranulomatous inflammation can be observed in several organs such as the kidneys or the gallbladder, its presentation in the female genital tract is rare, with the endometrium being the site where its presence has been most reported [7]. Involvement of the uterine tube is much less frequent, and this presentation has been associated with foci of tubal endometriosis [8], as observed in our first case, in which the first patient also presented with adenomyosis.

The etiology of this disease is not clear yet, but the infection theory (pelvic inflammatory disease) is one of the most accepted theories since, in many cases, the presence of certain pathogenic microorganisms such as *Escherichia coli*, *Bacteroides fragilis*, *Proteus*, and *Salmonella* has been demonstrated [9,10]. Nonetheless, some cases have been reported as without being related to infectious processes [11]. Additionally, a strong association with cervical stenosis has also been observed, which could cause a pyometra that would lead to xanthogranulomatous inflammation.

The classic radiological findings of this entity are usually a heterogeneous uterine collection suggestive of pyometra or hematometra [12], as in our second case in which the imaging diagnosis coincided with the pathology report of the endometrial biopsy. However, the images in some cases may be suggestive of other benign or malignant pathologies, as in the first case presented where the involvement of the entire uterine wall surpassed the serosa and compromised the omentum and the bladder serosa.

The aforementioned shows that XGE is a mimicker of different endometrial pathologies (with endometrial carcinoma being the most serious), as its clinical-radiological findings are nonspecific; therefore, it should be included in the differential diagnosis of diseases that present with vaginal discharge, such as bacterial infectious endometritis (e.g., tuberculosis endometritis), endometrial cancer, and endocervical cancer, three of the most prevalent diseases that hit low-income countries [13].

Additionally, the diagnosis of XGE does not necessarily rule out the presence of neoplasia. XGE has been observed in certain cases in association with endometrial hyperplasia or carcinoma [5]. Consequently, a complete curettage of the uterine cavity (exhaustive sampling) is recommended to determine the entity we are dealing with and rule out the presence of these associated pathologies [14].

The management of XGE is still controversial. According to current literature, in most previously reported cases, there was a complete recovery of the patient after the administration of antibiotics or even a spontaneous resolution. However, in some cases, surgery was required due to a lack of response to conservative treatment or the risk of systemic inflammation that could lead to death [15].

The two cases that we have presented represent extreme spectrums of the same pathology; on one hand, we could have a patient with a classic presentation of endometritis from the clinical, imaging, and pathological point of view, while on the other hand, we could also face a diagnostic challenge when the presentation simulates an endometrial carcinoma.

## Conclusions

Xanthogranulomatous endometritis is a very unusual pathological entity that mimics benign and malignant pathologies (such as endometrial carcinoma) very well. An inadequate diagnosis can lead to unnecessary surgeries, putting our patients' lives at unnecessary risks. Knowledge of this unusual and rare pathology, including its etiopathogenesis, is important since it can be included in multiple differential diagnoses of diseases that present with vaginal discharge; therefore, histopathological examination is essential to establish the diagnosis and, whenever it is found, avoid excessive treatment, decreasing the rate of surgical complications and health costs.
